# Contrast-Enhanced Mammography: A Literature Review of Clinical Uses for Cancer Diagnosis and Surgical Oncology

**DOI:** 10.3390/cancers16244143

**Published:** 2024-12-12

**Authors:** Wai-Shan Chung, Ya-Chun Tang, Yun-Chung Cheung

**Affiliations:** 1 Division of Breast Surgery, Department of Surgery, Chang Gung Memorial Hospital, Taoyuan 33305, Taiwan; m1124@cgmh.org.tw; 2 Department of Medical Imaging and Intervention, Chang Gung Memorial Hospital, Medical College of Chang Gung University, Taoyuan 33382, Taiwan; n884121@yahoo.com.tw

**Keywords:** mammography, contrast-enhanced mammography, breast cancer, cancer diagnosis, breast surgery

## Abstract

Contrast-enhanced mammography (CEM) uses intermittent dual-energy (low- and high-energy) exposures to produce low-energy mammograms and recombine enhanced images after the administration of iodized contrast medium, which provides more detailed information to detect breast cancers by using the features of morphology and abnormal uptake. In this article, we reviewed the literature to clarify the clinical applications of CEM, including (1) the fundamentals of CEM: the technique, radiation exposure, and image interpretation; (2) its clinical uses for cancer diagnosis, including problem-solving, palpable mass, suspicious microcalcification, architecture distortion, screening, and CEM-guided biopsy; and (3) the concerns of surgical oncology in pre-operative and neoadjuvant chemotherapy assessments. CEM undoubtedly plays an important role in clinical practice.

## Simple Summary

Contrast-enhanced mammography is a modern mammographic examination simultaneously using low- and high-energy exposures after the administration of iodized contrast medium to obtain a low-energy mammogram and a recombined enhanced image, providing precise information for detecting and diagnosing breast cancers by using morphology and the abnormal uptake of iodized contrast medium. Clinical applications have been investigated and reported since the approval of CEM in 2012. As its availability increases in daily clinical practice, clinicians should understand this emerging breast imaging modality. This review aims to present the uses for screening, cancer diagnosis, and surgical assessments. CEM undoubtedly have high clinical accessibility and practical feasibility.

## 1. Introduction

Contrast-enhanced mammography (CEM) is a modern mammographic examination simultaneously using low- and high-energy exposures after the administration of iodized contrast medium to obtain a low-energy mammogram (LM) and a recombined enhanced image (REI). The images provide precise information for detecting and diagnosing breast cancers by using morphology and the abnormal uptake of iodized contrast medium. Since the first approval of its clinical use in 2012, its clinical applications have been widely investigated in certain scenarios and the increased frequency of clinical uses. The clinicians essentially need to know the knowledge for screening, cancer diagnosis, and surgical assessments.

In this article, we reviewed the published results regarding CEM’s clinical uses and assessed their practicality in relation to the following three aspects:

(1) To understand the fundamentals of CEM including the technique, radiation exposure, and image interpretation;
(2) To familiarize ourselves with the clinical uses for cancer diagnosis including for problem-solving, palpable mass, suspicious microcalcifications, architecture distortion, screening, and CEM-guided biopsy; and
(3) To consider the clinical applications for pre-operative planning and neoadjuvant chemotherapy response assessments.

## 2. Fundamentals of CEM

### 2.1. CEM Technique

CEM employs dual-energy exposures on a conventional mammography system to obtain pairs of LMs and an REI. The technique utilizes the photoelectric effect to highlight the area of contrast medium uptake. CEM uses X-ray energies just below and above the K-edge of iodine (33.2 keV) to generate paired low- and high-energy mammographic images. Subsequently, the computer masks the different attenuations on these two low- and high-energy images and suppresses noise from non-enhanced breast structures to generate an REI. The REI shows the interstitial accumulation of iodine, indicating the contrast enhancement. The LM is ostensibly comparable to a full-field digital mammogram (DM) [1,2]. Both LM and REI are used for image interpretation.

**Figure 1:**
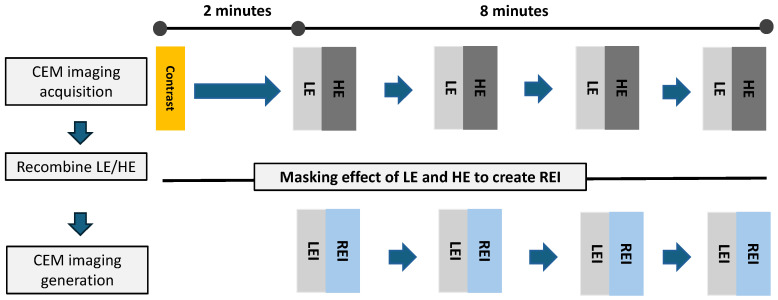
Diagram of dual energy contrast enhanced mammography protocol. Two minutes after the administration of the contrast medium, low- and high-energy images of both breasts are obtained within the next eight minutes. Subsequently, low-energy and recombined images are acquired through post-imaging processing for clinical diagnosis. LE = low energy, LEI = low-energy image, HE = high energy, REI = recombined imaging.

The administration of iodized contrast medium is advised at a flow rate of 3 mL/s, with a total dose of 1.5 mL/kg of body weight, via an intravenous catheter inserted into the forearm. To allow for the flow of contrast medium into the breasts, an examination with breast compression is started with intermittent exposures of low and high energy 2 min after injection. As in conventional mammography, craniocaudal (CC) and mediolateral oblique (MLO) views of the bilateral breasts are obtained. The CEM acquisition is suggested within 2 to 10 min after the injection of contrast medium to ensure optimal perfusion of the breast tissue [3]. The performance is shown in Figure 1. Although the contrast enhancement may remain visible for up to 10 min, the visualization of enhanced cancer may be interrupted by the superimposed fibroglandular enhancement in the late acquisition. This is because the enhancement of the suspicious region and breast parenchyma has already started to dynamically change once the contrast medium has been injected. Radiologists and technicians need to be aware of the enhancement–time relationship and the need for a smooth performance of the patient positioning.

The administration of iodized contrast medium is essential for CEM. Women with renal function impairment, hyperthyroidism, pregnancy, lactation, or a history of allergic reactions are prohibited from undergoing the examination. Allergic reactions potentially happen with an incidence of about 0.8% and are mostly mild and self-limited. A severe allergic reaction is still possible, with a low incidence of 0.007% [4]. It is imperative that all of the staff involved in conducting CEM pay attention to the status of the patient and understand how to treat these reactions.

### 2.2. CEM Radiation

Each acquisition in CEM consists of intermittent low- and high-energy exposures, leading to a higher radiation dose than that in DM. Several studies have evaluated the radiation dose associated with CEM as compared to conventional DM and 3D tomosynthesis. Hendrick et al. [5] observed that the radiation dose for CEM is 20–45% higher than that for traditional 2D mammography. Nicosia et al. [6] found that the median average glandular dose (AGD) from CEM was significantly lower compared to that from digital mammography with one projection of digital breast tomosynthesis. Several studies have also investigated the relationship between the radiation dose of CEM examinations and patient-specific factors. A study compared the radiation dose of CEM protocols between different breast thicknesses and compression forces in the same patients and concluded that only breast thickness could influence the AGD [7]. Another study even reported that 173 patients with dense breasts had lower radiation exposure than those with non-dense breasts during CEM examinations [8]. Despite the increased radiation dose associated with CEM, the AGD value for each projection complies with the Mammography Quality Standards Act regulations [9]. Concerns about the radiation dose are acceptable and appear not to impede the future of clinical implementation.

### 2.3. Image Interpretation

CEM results in a kind of morpho-functional image obtained by analyzing the LM and REI in the same session of patient positioning during the examination. The additional information from the REI improves breast cancer detection and diagnosis. The interpretation of LMs and conventional DMs is basically the same in clinical practice for radiologists and breast surgeons. When reading REIs, the abnormal enhancement can be easily identified and alert the reader due to its association with malignancies. Unfortunately, the breast tissues can be physiologically enhanced to become a parenchymal background. The changes in parenchymal enhancement have been explained as secondary to the phase of the menstrual cycle. This impact has been reported as minimal regarding the extent of parenchyma enhancement, which does not interfere with the result [10,11]. Furthermore, it is important to note that most breast cancers are enhanced faster and stronger than ordinary perfused breast tissues because of cancer angiogenesis.

Understanding breast parenchymal enhancement is the first critical step in interpretation. The enhancement extension is simply reported as minimal, mild, moderate, or marked, similar to the current descriptors used from the magnetic resonance imaging (MRI) Breast Imaging Reporting and Data System (BI-RADS) lexicon. Like breast density on a mammogram, marked enhancement of the parenchymal background may potentially mask or superimpose the cancer and influence the cancer sensitivity. When a suspicious enhancement is observed, both the LM and REI need to correlate mutually. Other image examinations, such as tomosynthesis, spot compression mammography, or sonography, may be used to identify any detectable morphologic lesion. When no morphologic lesion is found, enhanced MRI may be used to confirm the presence of a suspicious enhancement. Currently, CEM-guided biopsy has been developed for histologic diagnosis, exclusively for such cases of suspicious enhancement only.

The false negatives at CEM can certainly result in missed breast cancers and delay necessary treatment. To our knowledge, there is a lack of research on the false negatives. The assessment is hard due to a long-time survey. However, the reasons for false negatives include the incomplete or inadequate visualization of a lesion, background parenchymal enhancement obscuring lesion enhancement, a lack of lesion enhancement, or errors of characterization by the interpreting radiologist. The incomplete visualization resembles DM because of the improper positioning or lesions at the borderland. To facilitate the recognition of suspicious enhancement, we therefore standardized the protocol to obtain bilateral breasts for comparison in the approximate time zones (CC view within 2 min to 3 min and MLO view within 3 min to 6 min). Enhanced MRI may further evaluate the suspicious cases without significant enhancement or clarify the characters of the lesions.

Generally, the same view is alternated between the bilateral breasts so that at least one view of each breast is obtained while contrast is maximally present (within 3 min after injection). Although the in-charge radiologists can have the right to design the examination protocol in different institutions according to their clinical needs or research, they need to clearly describe the details of performance and image findings in the report in order to lower the influence on the reproducibility.

## 3. CEM for Cancer Diagnosis

### 3.1. Problem Solving

Breast cancer is the most common fatal disease among the female population worldwide, accounting for 24.2% of all cancers [12]. However, the five-year relative survival rate can significantly increase when breast cancer is detected early as a localized disease [13]. Mammography is the most cost-effective imaging modality for breast examination and is recognized as the best way to detect early-stage breast cancer and reduce mortality from this disease through screening investigations [14,15,16,17]. The overall cancer sensitivity with mammography has been reported to range from 70% to 80% [18,19,20]. Unfortunately, cancer sensitivities vary in different breast densities, decreasing from 98%, 83%, and 64% to 48%, respectively, in fatty, homogeneous scattered glandular tissue, heterogeneously dense breasts, and extremely dense breasts [21]. This limits the evaluation of dense breasts. Many reports of CEM in dense breasts have been published, some of which are listed in Table 1 [22,23,24,25,26]. All of the reported sensitivities and specificities of CEM are higher than those of DM. Additionally, Lin et al. [27] reviewed 985 lesions from 10 studies and reported 95% sensitivity and 81% specificity. Although many benign breast abnormalities such as fibroadenoma, papilloma, radial scar, ductal hyperplasia, and inflammation/infection can also be enhanced, the diagnoses can ultimately be proved using biopsy [28].

In 2022, the BI-RADS lexicon for CEM was published as a supplement [29], basically using the BI-RADS lexicons of mammography and MRI to interpret the findings of LMs and REIs. Although many worldwide clinical data and experiences have been collected since the approval of CEM for clinical use in 2012 [3], the current CEM lexicon is still an early work on this novel technique.

The diagnostic performance of CEM compared to other conventional breast modalities has been investigated. For daily clinical work, we need to know, first, how well the REI solves the problems of the common mammographic abnormalities of mass, microcalcifications, and architecture distortion, and, second, the benefits of using CEM in a screening population.

### 3.2. Palpable Mass

A palpable mass is a frequent complaint of a woman visiting the breast clinic or outpatient department of a hospital. The appearance of a mammographic mass is a 3D configuration with a bulging edge, and the finding mostly represents the presence of a tumor. The detection of a tumor depends on its size and the background of breast density in which the overlapping glandular tissue can interrupt the sensitivity and differentiation. Data from a Dutch report on CEM established that 152 of 199 screening recalls (76%) were masses, and CEM increased the cancer sensitivity from 93% to 96.9% and the specificity from 35.9% to 69.7% compared to conventional mammography [30].

**Figure 2:**
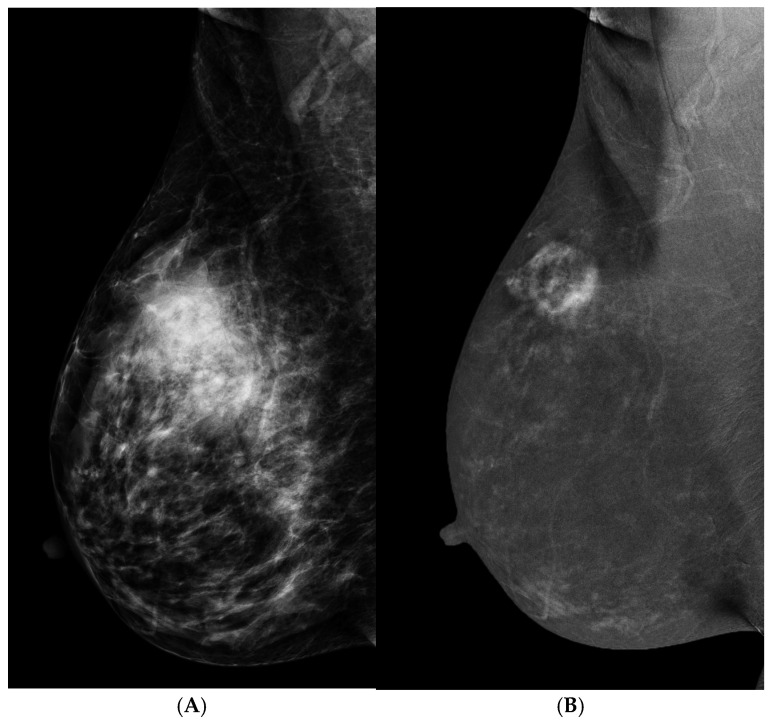
A 47 y/o woman with palpable mass in the upper outer quadrant of the right breast. (**A**) LM (MLO view) showed irregular hyperdense asymmetricity in the upper region of the right breast. (**B**) REI (MLO view) revealed a round cavitary enhanced mass with irregular and thick rim enhancement, as well as the presence of internal enhancement. It was surgicohistologically diagnosed as invasive ductal carcinoma.

**Figure 3:**
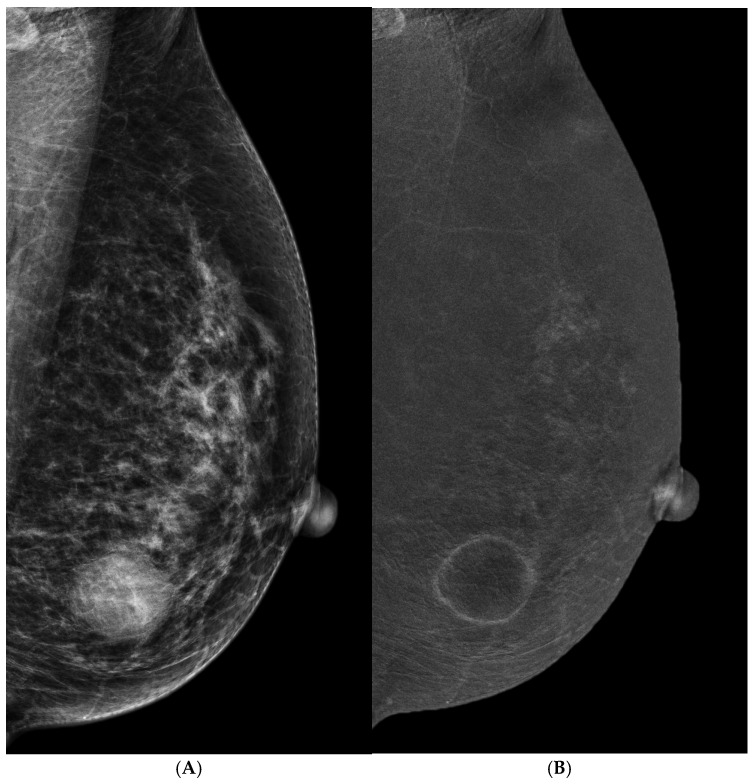
A 52 y/o woman with a palpable mass in the lower inner quadrant of the left breast. (**A**) LM (MLO view) showed a round circumscribed hyperdense mass in the lower region of the left breast. (**B**) REI (MLO view) showed a thin and smooth rim enhancement without internal enhancement, which was compatible with a simple inflamed cyst. The sonography of the breast also demonstrated a pure simple cyst.

Both malignant and benign tumors can be enhanced. When an enhanced mass is found, the REI features provide valuable information to determine the shape and outline of masses. An irregular shape or outline of masses is the strongest feature of breast cancers, which is similarly observed in other imaging modalities. A smooth outline of oval or round masses is the most common sign of a benign tumor. Rim enhancement has been reported as a highly suggestive feature for breast cancers [31,32], particularly with asymmetric thick or nodular rim enhancement (Figure 2). A thin rim enhancement without any internal density in a circumscribed mass may be a simple inflamed cyst (Figure 3). When the internal enhancement seems low, sonography or enhanced MRI is essentially needed for the better internal characterization of cystic, necrotic, central fibrosis, or low-angiogenic cancer components. For these sonographic observable masses, sonographically guided biopsy is warranted.

There have been no studies conducted to specifically compare the characterization of masses across DM, sonography, CEM, and enhanced MRI. CEM’s mass characterization ability is expected to be weaker than that of enhanced MRI or sonography, which both provide cross-sectional images to observe the internal or outline features of the entire tumor. Nevertheless, CEM plays an important role in displaying cancers and suspicious lesions. The cancer sensitivities have been reported as superior to those of DM [33] and sonography alone [34] and approximate to those of DM plus sonography [35] and enhanced MRI [36]. While CEM may not play a significant role in characterizing breast masses, its ability of cancer detection for the screening, abnormality evaluation, and cancer staging of multifocal, multicentric, or contralateral disease remains useful. A recent review published a ‘pro-CEM’ perception of the arguments for why breast MRI is hardly necessary when CEM in combination with sonography can be performed as a single-appointment imaging strategy in breast cancer patients [37]. Otherwise, the information on LM and REI in a single examination is practically more comprehensive than that on conventional DM.

### 3.3. Suspicious Microcalcifications

**Figure 4:**
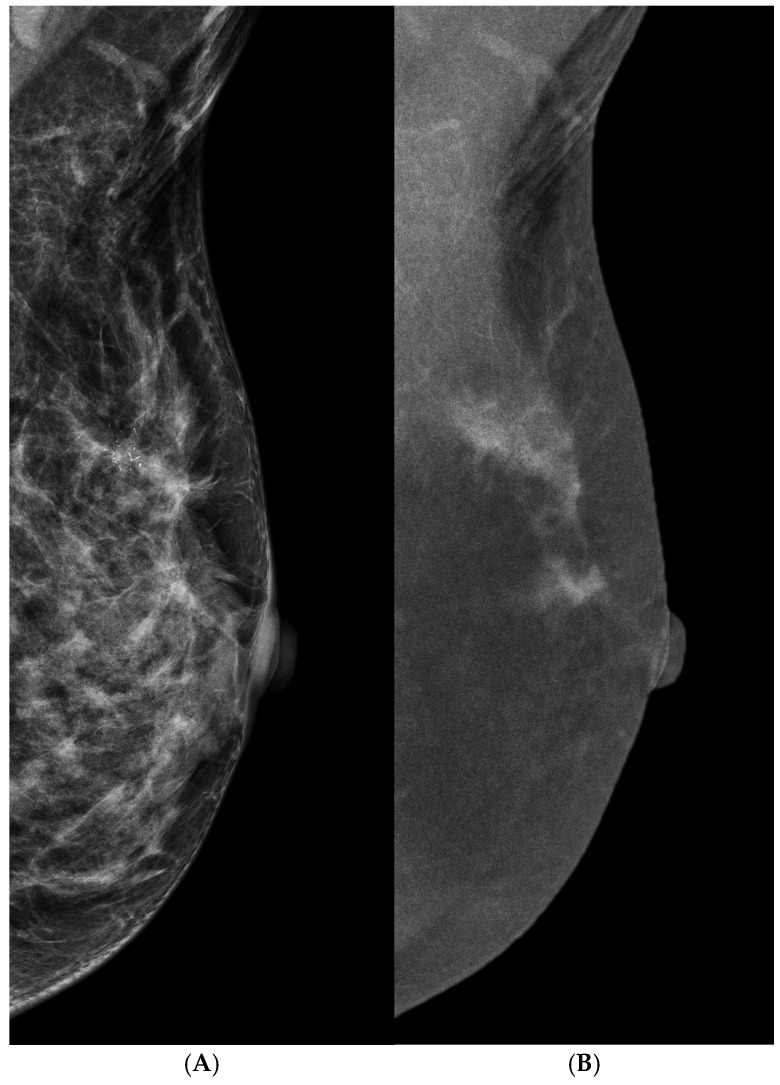
A 52 y/o asymptomatic woman with suspicious microcalcifications in the left breast by mammographic screening. (**A**) LM (MLO view) showed a group of amorphous microcalcifications in the upper outer quadrant of the left breast. (**B**) REI (MLO view) displayed an irregular segmental enhancement in the upper outer quadrant of the left breast. The extension of enhancement obviously seemed larger than the microcalcifications. Finally, it was surgically proven to be invasive ductal carcinoma.

Microcalcifications are common findings discovered via mammography that may be malignant, high-risk, or benign lesions. Using DM, many suspicious microcalcifications without associated masses are discovered and eventually diagnosed as early breast cancers. Most of them are noninvasive ductal carcinoma, and the prognosis is excellent with local excision control. Cancer diagnostic rates of about 20–25% with mammographic-guided needle biopsy have been reported [38,39,40,41]. However, concerns about overdiagnosis or over-performance have been raised. With CEM’s advantage of evaluating the morphologies and distributions of suspicious microcalcifications, associated or not with enhancement by the mutual correlation of LM and REI (Figure 4), many studies have reported its diagnostic performance on suspicious microcalcifications (Table 2) [42,43,44,45,46]. Cheung et al. [42,43] reported a high-sensitivity and negative predictive value (NPV) of CEM for microcalcification assessment and subsequently identified a higher positive predictive value (PPV) for pleomorphous microcalcifications with enhancement than for amorphous microcalcifications (90% vs. 46.15%) from recalls in mammographic screening. Depretto et al. [47] reported that the diagnostic performance of CEM using a deep learning model had similar results of a significantly higher NPV for low-suspicious microcalcifications as for intermediate–high microcalcifications (98% vs. 57%). These suspicious microcalcifications with low-risk appearances and a lack of enhancement may indicate benignity rather than a cancerous status. Clinically, a large number of vacuum-assisted biopsies of benign microcalcifications are often requested because of the risk of misinterpretation of suspicious microcalcifications. The over-performance of unnecessary biopsies can be reduced by CEM. Eventually, the patients need to understand the requirement of a follow-up strategy. For those with enhancement, the possibility of cancer increases and biopsy must be pursued.

### 3.4. Architectural Distortion

Architectural distortion (AD) is a commonly overlooked mammographic finding that can relate to various benign or malignant entities. A study found that the PPV of cancer using mammography was 74.5% among 369 cases of AD [48]. A review of 857 ADs using tomosynthesis from 13 retrospective observational studies yielded a pooled PPV for malignancy of 34.6% [49]. Of the 46 excised ADs detected by means of tomosynthesis without sonographic correlation, 47.8% were finally proven to be cancers [50]. Further evaluation is thus recommended. Similar to enhanced MR, CEM also characterizes lesion enhancement based on the principle of the presence of underlying pathologies and the absence of benign entities. Patel et al. reported that CEM’s sensitivity and NPV for cancers were 96.7% and 91.7%, respectively [51], which implies the usefulness, despite the risk, of stratifying for biopsy. Further studies using enhanced MRI or tomosynthesis may be considered before the decision to perform a biopsy is made.

### 3.5. Screening

Mammography has been accepted as the standard for breast cancer screening in the average female population. For populations with an intermediate or high risk (>20%) of breast cancer during their lifetime, enhanced MRI is recommended as a supplemental screening method to improve cancer detection. However, associations have been found with increased false-positive rates, unnecessary biopsies, and increased costs [52,53,54]. CEM is thus considered to be another option for cancer screening while preserving sensitivity. Its additional benefits include its high sensitivity for microcalcifications and the highlighting of enhanced masses on LM or REI, which is familiar to all breast radiologists. Nevertheless, enhanced MRI is still the priority for high-risk patients, particularly for those allergic to iodized contrast medium. Conversely, those patients who cannot receive MRI should be recommended for CEM.

Jochelson et al., in their study of 1200 women with an intermediate or high risk of breast cancer, found an approximate PPV of 20.9% in both CEM and enhanced MRI [55]. This provided insights into the use of CEM for primary and supplemental screening. In comparisons of CEM with enhanced MRI, the cancer sensitivity was similar, but the false-positive rate was lower [56]. With the preservation of approximate performance, the cost savings of using CEM instead of enhanced MRI were estimated at USD 1.1 billion annually [54], making it more cost-efficient. The shorter examination time, greater machine availability and patient comfort, and lower image reading requirements are also practical advantages of the use of CEM for screening. In fact, many investigations have documented the superior diagnostic performance of CEM on recalls from screening populations [30,57].

### 3.6. CEM-Guided Biopsy

**Figure 5:**
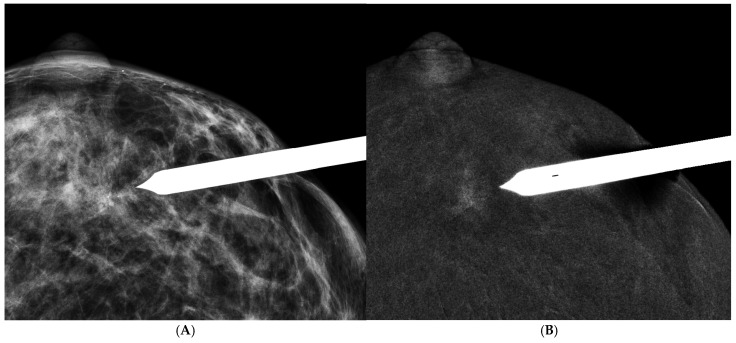
CEM-guided biopsy with the horizontal approach of a biopsy needle. (**A**) LM (CC view) showed a biopsy needle near the isodense suspicious lesion. (**B**) REI (CC) confirmed the correct locations of the needle and target. The biopsy was then fired through the target for the biopsy. The biopsy and subsequent surgery diagnosed it as ductal carcinoma in situ.

Recently, CEM-guided biopsy (CEM-Bx) has been commercialized for the histologic diagnosis of abnormal enhancements [58]. Clinically, targeted second-look sonography is often adopted for further evaluations when a suspected lesion is found via CEM. Coffey et al. [59] reported that only 31% of CEM-detected lesions could be morphologically identified with targeted second-look sonography. In cases without an obvious morphological target in mammography or sonography, the implementation of CEM-Bx essentially fills the diagnostic gap left by CEM. CEM-Bx is always an option when another modality displays the same suspicion as CEM; however, CEM-Bx is exclusive to cases identified solely by CEM (Figure 5). Many studies have reported the outcomes of CEM-Bx and concluded that this technique is feasible for diagnosing enhanced lesions on CEM [60,61,62]. CEM-Bx is an alternative to MRI-Bx for an enhanced suspicious lesion [63,64]. The benefits of using CEM-Bx instead of MRI include its lower cost, better machine availability, shorter procedural time, and greater practicality for breast radiologists.

Although CEM-Bx is a simple procedure, over-performance should be avoided to achieve better cost-efficiency. Several studies have investigated the possibility of reductions in unnecessary biopsies by using CEM. Amir et al. [65] reviewed 26 true-positive lesions and 147 false-positive lesions. A true-positive result was more likely (*p* = 0.02) for lesions present on both LM and REI (31%) than on LM only (4%) or REI only (12%). Among the lesions present on both low-energy and iodine images, a true-positive result was more likely (*p* < 0.001) when the type of mammographic finding was microcalcification (80%) than when it was a mass (11%) or distortion (0%). The presence of corresponding CEM findings with either sonography or enhanced MRI was more likely to be associated with a true positive than the absence of such findings. Among 25 false-positive calcifications, 24 had no associated mammographic enhancement. Grażyńska et al. [66] analyzed 528 patients classified according to BI-RADS 4 CEM with core needle biopsy, resulting in NPVs of 100% for mass lesions, 97.8% for non-mass lesions, and 87.9% for microcalcifications. About 60% of unnecessary CNBs could been correctly avoided among the 230 of 383 benign lesions without enhancement.

LM and REI should be mutually correlated for every case. The presence of enhancement may be associated with cancers, but it is not absolute; otherwise, the lack of enhancement is suggestive of a benign tumor. This information is valuable for reducing unnecessary biopsies. It is unknown whether unenhanced malignant microcalcifications relate to the issue of overdiagnosis.

## 4. CEM for Surgical Oncology

### 4.1. Operation Assessment

Breast-conserving surgery is preferable to total mastectomy in the treatment of breast cancers for better post-operative recovery and quality of life. CEM’s applications in pre-operative assessments concern (1) identifying the size and location of the cancer for breast excision and planning oncoplastic reconstruction; (2) detecting multifocal, multicentric, or contralateral cancers for complete curative surgical treatment; and (3) evaluating the cancer response after neoadjuvant chemotherapy.

In terms of the cancer size, it is difficult to assess periductal and peritumoral infiltrating cancer extensions morphologically using conventional breast images, particularly in cancers of a non-mass type or with large-extent suspicious microcalcifications [67,68]. Enhanced MRI is the best way to assess the cancer size [69,70]. Although size overestimations have been reported, MRI does provide benefits for surgical planning [71,72]. Many reports have also compared CEM to enhanced MRI in pre-operative settings, resulting in comparable sensitivities [73,74] and higher specificity [75,76]. Goh et al. [77] reported that surgical plans were pre-operatively altered for 36 of 200 patients (18%) after the use of CEM. Among them, the operation plans were changed to total mastectomy in 67% of patients due to a larger cancer size (>2 cm) and 33% of patients due to additional cancers.

**Figure 6:**
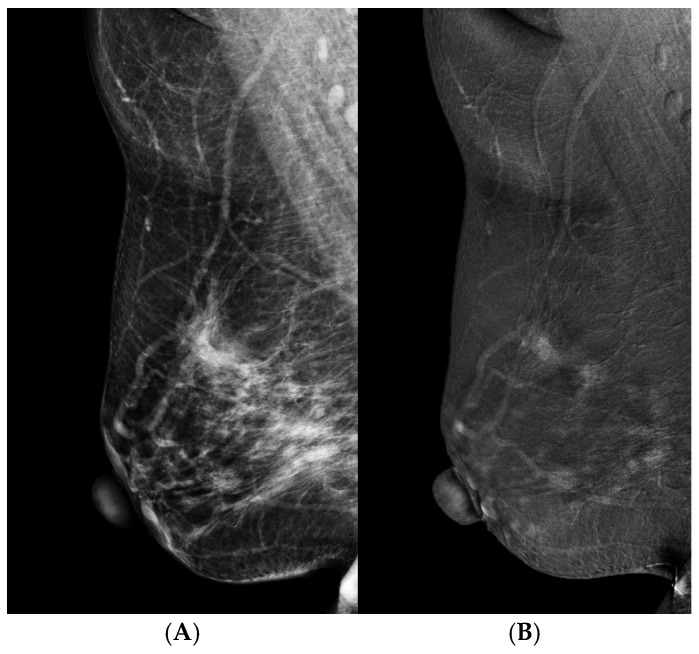
A 53 y/o women with a palpable mass in the upper outer quadrant of the right breast that was diagnosed as a papillary tumor by sonographic guided biopsy. (**A**) LM (MLO view) showed an irregular hyperdense mass in the upper region of the right breast and a nodular lesion with suspicious microcalcifications in the lower region of the right breast. (**B**) REI (MLO view) revealed multiple enhanced masses in the upper and lower regions of the right breast. The total mastectomy finally documented multicentric invasive ductal carcinomas.

The complete removal of multifocal (nearby, in the same quadrant) or multicentric (in a different quadrant) cancers is essential for a complete curative mastectomy (Figure 6). The prevalence of multifocal and multicentric cancers varies from 14% to 47% according to different imaging methods [78]. The presentation of multiple cancers is associated with a higher incidence of local recurrence [79]. Therefore, all cancers or foci, either invasive or non-invasive, need precise detection for adequate excision. However, multifocal cancers are frequently morphologically small or are infiltrating. The whole territory of the cancer is essentially measured for cancer excision. When the cancer-to-breast size ratio is too large, total mastectomy is preferable to conservation mastectomy. The detectable cancer extent is the basic information required for adequate volume excision with a safe margin, as well as for oncoplastic reconstruction. Breast-conserving surgery is preferable to total mastectomy, as reported for equivalent or even more beneficial survival in the long term [80,81,82,83,84,85]. However, total mastectomy was still indicated in the patients with extensive or multifocal diseases. Otherwise, the information of cancer extension and focality has been documented to be the major predictors of mastectomy after neoadjuvant chemotherapy [86]. Contrast images, including CEM and MRI, have been proven to enhance the detection of focality and the extension of cancers that may be easily missed on standard pre-op images.

CEM can serve well as a surveillance follow-up exam in detecting locoregional minimal cancer recurrence after breast surgery [79]. Helal et al. [87] concluded that CEM is a credible technique for detecting malignancy in the post-operative breasts of cancer patients, with a sensitivity of 91.17%, a specificity of 75%, a positive predictive value of 77.5%, and a negative predictive value of 90%.

### 4.2. Neoadjuvant Chemotherapy Response Assessment

CEM is a possible imaging tool for the assessment of treatment responses. Recently, neoadjuvant therapy has been widely employed for treating early-stage breast cancer in daily practice. Pre-operative cancer shrinkage provides the advantages of breast tissue preservation, drug response assessment, and adjustment of the following adjuvant therapy [88,89]. Physical examinations and breast imaging are used to evaluate the therapeutic response by monitoring the changes in the key index cancer during the treatment [90]. For palpable lesions, physical examinations can provide an assessment of the treatment response, especially in a concentric tumor shrinkage pattern. However, this method is not sufficiently sensitive to detect small or infiltrating viable cancers. The main cancers can divide into foci or subside into infiltrations, either concurrently with or without scatter microcalcifications that are hard to estimate using physical examinations, DM, or sonography [91]. Bernardi et al. prospectively compared CEM to enhanced MRI in predicting the complete response, showing no significant difference in sensitivities [92]. In other retrospective studies, CEM and enhanced MRI had approximately equal sensitivities and specificities, with 80% correct predictions [93,94,95].

Evaluating the residual cancer extent is important for surgical excision after neoadjuvant chemotherapy. Studies have compared the outcomes of CEM and enhanced MRI in estimating the residual cancer size. Sunen et al. [95] reported that the size overestimation by CEM was 2.87 mm, and that by MRI was 0.51 mm. Lotti et al. [96] reported that the residual tumor size underestimation with CEM was 4.1 mm, and that with MRI was 7.5 mm. This underestimation can be explained by the degrading effect of invasive to non-invasive components and the anti-angiogenesis effects of chemotherapeutic drugs. Another study suggested that delayed CEM acquisition at 6 min after contrast material injection is the best way to detect additional residual DCIS in the affected breast after neoadjuvant therapy [92]. The issue is how to distinguish residual cancers from delayed-enhancement glandular tissues. In fact, variations in size measurements within 10 mm are mostly assumed to be acceptable for surgical practice.

### 4.3. Future Prospects

CEM was developed a decade ago as a diagnostic imaging tool that is clinically applicable to cancer diagnosis, pre-operative evaluations, drug response monitoring, and biopsy guidance. Artificial intelligence (AI) for cancer diagnosis is the next stage; however, it is still under investigation. There are publications integrating CEM with an AI model that reported clinical breast cancer diagnosis with 80–90% negative predictive value and approximately 90% accuracy [97,98]. Currently, there is no AI model for commercial CEM in the market. CEM has already improved cancer visualization, in which AI tends to establish enhanced cancer and non-cancer differentiation. Otherwise, CEM with AI, combined with other radiomics and biochemistry, could even be applied to preventive medicine, personalized medicine, and pharmacokinetics discovery in the future.

## 5. Conclusions

The performance and cancer diagnosis among DM, sonography, CEM, and enhanced MRI have been compared in Table 3. CEM is a modern morpho-functional breast image modality that has been investigated and found to have high clinical accessibility and practical feasibility, not only for screening and cancer diagnosis but also for treatment response evaluations and surgical planning. Its cost-effectiveness is deemed superior to that of conventional mammography and not inferior to that of enhanced MRI.

## Figures and Tables

**Table 1:** Publications on the sensitivity and specificity of CEM in dense breasts.

Authors [Ref] (Pub Yr)	No.	CEM		Mammography	
		Sensitivity	Specificity	Sensitivity	Specificity
Cheung et al. [22] (2014)	89	93	68	72	52
Sorin et al. [23] (2018)	611	91	76	52	91
Azzam et al. [24] (2020)	37	89	89	83	48
Rudnicki et al. [25] (2021)	121	100	33	-	-
Moffa et al. [26] (2023)	110	93.5	80.9	79	47

Ref = reference; Pub Yr = publication year; No. = number of lesions.

**Table 2:** Publications reporting the sensitivity, specificity, PPV, and NPV of CEM for suspicious microcalcifications only.

Author [Ref] (Pub Yr)	No.	Sensitivity	Specificity	PPV	NPV
Cheung [42] (2016)	59	90.9	83.78	76.92	93.94
Cheung [43] (2016)	94	88.89	86.56	72.72	95.08
Houben [44] (2019)	147	93.8	36.6	54	88.2
Long [45] (2021)	74	77	88	77	88
Nicosia [46] (2023)	377	80	85.9	70.6	91

Ref = reference; Pub Yr = publication year; No. = number of lesions.

**Table 3:** Comparison of digital mammography (DM), sonography (Sono), contrast-enhanced mammography (CEM), and magnetic resonance imaging (MRI).

	DM	Sono	CEM	MRI
Performance				
Imaging mode	Morphology	Morphology	Morpho-functional	Morpho-functional
Radiation	Present	Absent	Present	Absent
Examination time	10 min.	15 min.	15 min.	30 min.
Contrast medium	No	No	Yes	Yes
Cost	Low	Low	Intermediate	High
Readily Available	Good	Good	Good	Not good
Potential side-effect	No	No	Allergy, Renal function loading	Allergy, Renal function loading, Psychological impact
Cancer Diagnosis				
Mass	Intermediate	Good	Good	Good
Non-mass	Intermediate	Intermediate	Good	Good
Microcalcification	Excellent	Poor	Excellent	Poor
Preferable role	Screening	Palpable mass	Suspicious lesions	Therapeutic assessment

## Data Availability

The datasets used and/or analyzed during the current study are available from the corresponding author on reasonable request.

## Abbreviations

CEM	Contrast-enhanced mammography
LM	Low-energy mammogram
REI	Recombined enhanced image
CC	Craniocaudal
MLO	Mediolateral oblique
DM	Digital mammography
AGD	Average glandular dose
MRI	Magnetic resonance imaging
BI-RADS	Breast Imaging Reporting and Data System
NPV	Negative predictive value
PPV	Positive predictive value
CEM-Bx	CEM-guided biopsy
AI	Artificial intelligence
